# Collecting and exploiting data to understand a nation’s sexual health needs: Implications for the British National Surveys of Sexual Attitudes and Lifestyles (Natsal)

**DOI:** 10.1136/sextrans-2018-053571

**Published:** 2019-03-19

**Authors:** Catherine H Mercer, Soazig Clifton, Gillian Prior, Robert W Aldridge, Chris Bonell, Andrew J Copas, Nigel Field, Jo Gibbs, Wendy Macdowall, Kirstin R Mitchell, Clare Tanton, Nick Thomson, Magnus Unemo, Pam Sonnenberg

**Affiliations:** 1 Institute for Global Health, University College London, London, UK; 2 NatCen Social Research, London, UK; 3 Institute of Health Informatics, University College London, London, UK; 4 Department of Public Health, Environments and Society, London School of Hygiene & Tropical Medicine, London, UK; 5 MRC/CSO Social & Public Health Sciences Unit, University of Glasgow, Glasgow, UK; 6 Department of Global Health and Development, London School of Hygiene & Tropical Medicine, London, UK; 7 Wellcome Trust Sanger Institute, Cambridge, UK; 8 WHO Collaborating Centre for Gonorrhoea and Other STIs, Örebro, Sweden; 9 National Reference Laboratory for Pathogenic Neisseria, Department of Laboratory Medicine, Microbiology, Faculty of Medicine and Health, Örebro University, Örebro, Sweden

**Keywords:** sexual behavior, sexual health, methodology, survey

Accurate information about a nation’s sexual health is essential to plan and evaluate services, inform prevention efforts, and contribute to societal discourse on sexuality. In Britain, sexual health data come from a range of sources. There are world-class surveillance data on STIs and reproductive health, although these only represent people attending services and collect limited data.^1 2^ A number of British surveys draw on convenience samples of populations of particular interest, such as sexual health clinic attendees, men who have sex with men and particular ethnic groups,^3–7^ and are therefore not generalisable to the wider population. Furthermore, such surveys often focus on specific aspects of sexual health (eg, STIs), and while some nationally representative surveys have included questions on sex and relationships, space limitations mean that questions are restricted to a small number of key behaviours and outcomes.^8^

Since 1990, the British National Surveys of Sexual Attitudes and Lifestyles (Natsal) have provided a decennial ‘census’ of the sexual health of the nation, capturing detailed data from probability samples of the population (Natsal-1: 1990–1991; Natsal-2: 1999–2001; Natsal-3: 2010–2012). This has involved randomly selecting addresses to which a trained fieldworker calls to randomly select and invite one household member to participate in a detailed structured interview using a combination of computer-assisted face-to-face and self-completion questions, with a subsample of participants invited to provide biological samples. This design seeks to represent the population as a whole, including those who do not access services or who would not usually volunteer to take part in a sex survey, for example, those who are not sexually active.

Natsal was initiated in response to the emerging HIV epidemic and has evolved to become internationally renowned in the population-based measurement of the social, behavioural and biological aspects of sexual health. Natsal has captured dramatic changes in sexual attitudes and lifestyles in Britain, such as earlier sexual debut, increasing partner numbers and same-sex experience.^9^ Natsal provides the evidence base for major sexual health interventions and monitoring their impact, including the National Chlamydia Screening Programme, enhanced HIV testing, HPV vaccination, the Teenage Pregnancy Strategy, and sex and relationship education.

As a decade has nearly passed since Natsal-3, new data are now needed to provide updated estimates, assess change over time, and shed light on contemporary topics, such as how digital technology impacts on how people learn about sex, meet partners and access services. Furthermore, with changes to sexual health service delivery and funding, updated evidence from Natsal is essential to evaluate consequences on outcomes and inequalities, commission future services, and examine the impact of ongoing and new initiatives.

It has recently been announced that a fourth wave of Natsal will be funded by Wellcome Trust as part of its Longitudinal Population Studies Strategy,^10^ with contributions from the Economic and Social Research Council (ESRC) and the National Institute for Health Research (NIHR). Wellcome’s new strategy follows the 2017 Longitudinal Strategic Review led by the ESRC.^11^ Both recognise that high-quality longitudinal studies—in their broadest sense—are essential for understanding how biological, social and environmental factors interact over time in populations to produce health outcomes.^10 11^ Yet they also acknowledge that such studies are expensive to run. For Natsal, a large part of the investment is spent on collecting the data, not least because of the increasing costs involved with undertaking interviewer-led, household-based probability sample surveys. As such, in designing Natsal-4 we revisited the study design and reviewed the potential methods for collecting reliable sexual health data and the extent to which they can meet the needs of data users and the wider sexual health community. Key considerations were the ability to represent the general population, provide adequate sample size, enable ‘boost’ samples of some population subgroups to ensure robust estimates (eg, young people and people from ethnic minorities), collect detailed information on a wide range of topics, collect biological samples, maintain data quality, request consent to link participants’ survey data to routine data sources, and be cost-efficient. We used these criteria to assess a number of options (table 1), relative to Natsal’s methodology to date (option 1). As part of our review, we gathered the latest evidence on the potential of mixed-mode surveys, including online components.^12^ However, a number of major issues still need to be overcome (eg, lower response rates, response bias, selection bias). The full scoping review, which includes the methods used by other population sex surveys internationally, is available from http://www.natcen.ac.uk/our-research/research/national-survey-of-sexual-attitudes-and-lifestyles-(natsal)/.

**Table 1:** Overview of design options considered for Natsal-4 and whether they meet data collection requirements based on available evidence

Option	Criteria							
	Cheaper delivery of data	Minimise sampling and response bias	Sufficient statistical power	Detailed questionnaire data including new topics	Young person/ethnic boost samples	Biological samples possible	Data linkage possible	Maintain time series
1. Probability sample + interviewer-led interview	NA	✓	✓	✓	✓	✓	✓	✓
2. Follow up existing probability sample survey	✕	(✕) Need to follow up for several years	(✓) Need to follow up several years.	✓	(✕) Depends on survey sample size.	✓	✓	✕
3. Add detailed sexual health module to existing survey	✓	✓	(✓) Need to include for several years.	(✕) Amount of detail depends on survey/sponsors.	(✕) Would need separate funding.	(✓) Depending on survey.	(✓) Subject to agreement from survey team.	(✓) For limited set of questions, depending on mode.
4. Probability-based web/telephone panel	**✓**	✕	✕	✕	✕	NK (✓)	NK (✕)	✕
5. Random digit dialling + telephone interview	✓	✕	✓	NK (✓)	NK	NK (✓)	NK (✕)	✕
6. ‘Web-first’ mixed-mode survey	NK	✕	✓	(✓) For some but not all participants.	NK (✕)	NK (✓)	NK (**✓**)	✕
7. Interviewer recruitment + web survey	NK (✓)	NK (✕)	✓	✕	✓	NK (✓)	NK (✓)	✕

‘(✓)’ indicates that this is possible, depending on the specific design chosen.

‘NK’ indicates insufficient evidence available from other surveys.

‘NK (✓)’ indicates insufficient evidence, but likely to be able to deliver or partially deliver.

‘NK (✕)’ indicates insufficient evidence, but unlikely to deliver.

NA, not applicable; Natsal, National Surveys of Sexual Attitudes and Lifestyles.

Given the drawbacks and uncertainty around cost-savings of the alternative options considered, we decided that Natsal-4 should retain the design used by previous Natsals. This also enables the rigorous assessment of trends, capturing generational changes and broad societal shifts through the measurement of both period and birth cohort effects. Looking to the future, we seek to collaborate with survey methodologists to undertake further research on how best to optimise a mixed-mode survey for sensitive topics like Natsal, which maximises response and minimises bias.

The shift across the funding landscape is also in terms of maximising investment, effort and impact.^10 11^ This requires researchers to be proactive in sharing their methods, data and other funded resources outside of the original research team, and to have mechanisms that will expedite this process, the objective being to enhance the quantity and range of outputs, their impact and reach, delivering added value not just for the individual study but longitudinal studies more broadly. For example, Natsal will expand the range of data on exposures, outcomes and potential confounders collected by the survey through linkage to health, administrative and geospatial data sets.

There will be many opportunities for the research community, policymakers, clinicians, educators and the public to engage with, and further exploit, the Natsal resource. This extends far beyond the biobehavioural data sets and includes questionnaires, fieldwork documentation, data user guides, validated measures and tools, educational materials and a biobank of residual samples. Natsal provides a platform for the wider research and educational communities to address specific policy/scientific questions and build capacity for the next generation of sexual health researchers in the UK and internationally.

## References

[1] Gov.UK Genitourinary Medicine Clinic Activity Dataset (GUMCADv2). https://www.gov.uk/guidance/genitourinary-medicine-clinic-activity-dataset-gumcadv2 [Accessed 17 Jan 2018].

[2] The National Archives, 2018 Sexual and Reproductive Health Activity Dataset (SRHAD) Collection. http://content.digital.nhs.uk/datacollections/srhad [Accessed 13 Feb 2018].

[3] Blood Borne and Sexually Transmitted Infections, 2017 Health Protection Research Unit. http://bbsti.hpru.nihr.ac.uk/ [Accessed 27 Oct 2017].

[4] Prah P, Hickson F, Bonell C, et al Men who have sex with men in Great Britain: comparing methods and estimates from probability and convenience sample surveys. Sex Transm Infect 2016;92:455–63. 10.1136/sextrans-2015-052389 26965869 PMC5013102

[5] Health Protection Agency, 2005 Mayisha II main study report. http://kwp.org.uk/files/mayisha_11_2005.pdf [Accessed 13 Feb 2018].

[6] Evans AR, Parutis V, Hart G, et al The Sexual Attitudes and Lifestyles of London's Eastern Europeans (SALLEE Project): design and methods. BMC Public Health 2009;9:399 10.1186/1471-2458-9-399 19878564 PMC2777871

[7] Gerver SM, Easterbrook PJ, Anderson M, et al Sexual risk behaviours and sexual health outcomes among heterosexual black Caribbeans: comparing sexually transmitted infection clinic attendees and national probability survey respondents. Int J STD AIDS 2011;22:85–90. 10.1258/ijsa.2010.010301 21427429

[8] Prah P, Johnson AM, Nardone A, et al Asking about sex in general health surveys: comparing the methods and findings of the 2010 Health Survey for England with those of the third National Survey of Sexual Attitudes and Lifestyles. PLoS One 2015;10:e0135203 10.1371/journal.pone.0135203 26252650 PMC4529206

[9] Mercer CH, Tanton C, Prah P, et al Changes in sexual attitudes and lifestyles in Britain through the life course and over time: findings from the National Surveys of Sexual Attitudes and Lifestyles (Natsal). The Lancet 2013;382:1781–94. 10.1016/S0140-6736(13)62035-8 PMC3899021 24286784

[10] Wellcome.UK, 2017 Longitudinal Population Studies Strategy Wellcome’s Longitudinal Population Studies Working Group. https://wellcome.ac.uk/sites/default/files/longitudinal-population-studies-strategy_0.pdf [Accessed 25 sep 2018].

[11] Longitudinal Studies Strategic, 2017 Report to the Economic and Social Research Council: executive summary and policy impact case studies. https://esrc.ukri.org/files/news-events-and-publications/publications/executive-summary-longitudinal-strategic-review-2017/ [Accessed 25 sep 2018].

[12] Villar A, Fitzgerald R Breen M, Using mixed modes in survey research: evidence from six experiments in the ESS. Europe: Values and Identities, 2017: 273–310.

