# Correction: *Collecting and exploiting data to understand a nation’s sexual health needs: Implications for the British National Surveys of Sexual Attitudes and Lifestyles (Natsal)*

**DOI:** 10.1136/sextrans-2018-053571corr1

**Published:** 2019-05-28

Mercer CH, Clifton S, Prior G, *et al*. Collecting and exploiting data to understand a nation’s sexual health needs: Implications for the British National Surveys of Sexual Attitudes and Lifestyles (Natsal). *Sexually Transmitted Infections* 2019;95:159–161.

The license type of the paper has changed from CC BY-NC to CC BY.

